# Investigating the Joint Amplitude and Phase Imaging of Stained Samples in Automatic Diagnosis

**DOI:** 10.3390/s23187932

**Published:** 2023-09-16

**Authors:** Houda Hassini, Bernadette Dorizzi, Marc Thellier, Jacques Klossa, Yaneck Gottesman

**Affiliations:** 1 Samovar, Télécom SudParis, Institut Polytechnique de Paris, 91120 Palaiseau, France; bernadette.dorizzi@telecom-sudparis.eu (B.D.); yaneck.gottesman@telecom-sudparis.eu (Y.G.); 2 TRIBVN/T-Life, 92800 Puteaux, France; jklossa@tribvn.com; 3 AP-HP, Centre National de Référence du Paludisme, 75013 Paris, France; marc.thellier@aphp.fr; 4 Institut Pierre-Louis d’Épidémiologie et de Santé Publique, Sorbonne Université, INSERM, 75013 Paris, France

**Keywords:** Quantitative Phase Imaging, Fourier Ptychographic Microscopy, complex-valued neural networks, malaria detection, *Plasmodium falciparum* detection

## Abstract

The diagnosis of many diseases relies, at least on first intention, on an analysis of blood smears acquired with a microscope. However, image quality is often insufficient for the automation of such processing. A promising improvement concerns the acquisition of enriched information on samples. In particular, Quantitative Phase Imaging (QPI) techniques, which allow the digitization of the phase in complement to the intensity, are attracting growing interest. Such imaging allows the exploration of transparent objects not visible in the intensity image using the phase image only. Another direction proposes using stained images to reveal some characteristics of the cells in the intensity image; in this case, the phase information is not exploited. In this paper, we question the interest of using the bi-modal information brought by intensity and phase in a QPI acquisition when the samples are stained. We consider the problem of detecting parasitized red blood cells for diagnosing malaria from stained blood smears using a Deep Neural Network (DNN). Fourier Ptychographic Microscopy (FPM) is used as the computational microscopy framework to produce QPI images. We show that the bi-modal information enhances the detection performance by 4% compared to the intensity image only when the convolution in the DNN is implemented through a complex-based formalism. This proves that the DNN can benefit from the bi-modal enhanced information. We conjecture that these results should extend to other applications processed through QPI acquisition.

## 1. Introduction

With the introduction of digitization, the healthcare sector has been undergoing a profound transformation for more than a decade. This transformation is primarily based on the availability of technologies that enable the digital acquisition of biological samples [1,2]. The expected benefits for patients are numerous. These include the remote transmission of data to experts for the management of rare situations, the establishment of complete and reliable automated diagnoses for the management of patients in critical situations, and the archiving of data for improved traceability of diagnosis and patient follow-up [3].

As microscopy remains a frequent first-line examination, hospital workflows are now well-suited to accommodate microscopy slide digitization solutions. Today’s scanners can generate complete virtual slides that can be navigated by computer. Once coupled with image analysis algorithms, they should enable automated diagnosis. However, developing highly sensitive and specific automated systems remains a delicate task, despite the already appreciable quality of the images delivered by actual scanners. To improve image-based automatons performances, several directions are under investigation. The first concerns the design of image analysis algorithms based on artificial intelligence techniques [4]. They can be adapted to the available data and further optimized when necessary. New Deep Neural Networks (DNN) approaches dedicated to biological samples are also regularly introduced [5,6,7].

A second direction concerns the acquisition of enriched information on samples. In particular, Quantitative Phase Imaging (QPI) techniques are attracting growing interest [8,9,10,11]. In addition to the intensity transmitted through the sample, as traditionally recorded in microscopy, the phase of samples is also recorded. This phase is an interesting additional information, since it is associated with the time it takes for light to propagate through the sample, i.e., its optical path. It thus represents data related to sample morphology along the z-axis. Depending on its exploitation, it can be used, for example, to measure the dry mass of cells or to represent the sample in the form of 3D tomographic cross-sections [12,13]. In addition, the used wavelengths that belong to the optical domain enable the observation of tiny variations in the sample in the nanometer range. In other words, these technologies are exciting because they give access to distinct interaction mechanisms between light and the sample, depending on the nature of the signal, intensity, or phase. In such a perspective, the light is seen as a probe. The recorded image then constitutes data from which interaction parameters can be extracted quantitatively [14].

Until now, these techniques have been used extensively to reveal fine spatial variations in samples [15,16]. They are also particularly suitable for observing transparent samples that are difficult to analyze with conventional microscopes [17,18]. Recent advances in in-line digital holography and computational microscopy make them much simpler to use, since they avoid the need for an interferometry set-up that is not fully adapted for routine sample examination. This renders these techniques compatible with hospital laboratory environmental constraints. In addition, QPI greatly facilitates sample digitization over large areas, enabling the production of complete virtual slides [19].

While the benefit of using phase images is quite evident for naturally transparent objects [20,21,22], the question remains open regarding its interest in stained sample analysis. More precisely, the role of the dyes employed in sample preparation is to reveal the cellular compartments of interest by selectively modifying their absorption properties with wavelength. The color variations consequently arising on the samples enable the specialist to identify the distinctive elements needed to establish a diagnosis from an intensity image only. In this sense, the image processing algorithm applied to colored-intensity images should already be sufficient to establish the desired automated diagnosis. Nevertheless, the benefits of recording phase images additionally to intensity images need to be investigated in greater detail for the following reasons: the images produced with QPI are quantitative. It is not the case with Köhler illumination, since multiple incident angles are used simultaneously to probe the sample. The absorption recorded at the camera plane results from the different light paths in the sample. The image thus recorded, therefore, provides only qualitative information on the local absorption of the sample (except possibly for infinitely thin objects).

Furthermore, phase images provide access to morphological information along the z-axis that is not accessible to traditional approaches. As a result, intensity and phase image fusion techniques will likely bring greater robustness to subsequent processing. Finally, it should be noted that the presence of dyes can also modify the phase. Through Kramer–Kronig relation [23], an enhancement of the cellular compartments of interest may also be expected in phase.

In this work, we study the contribution of joint intensity and phase image modalities in establishing image-based automatic diagnosis. We restrict ourselves to the *Plasmodium Falciparum* detection use case (useful for malaria detection) to address this general question [24]. The considered blood smear samples are stained with May–Grünwald Giemsa (MGG). This use case has been chosen since detecting infected red blood cells is a reasonably simple problem, which nevertheless remains challenging. Different implementations of a Convolutional Neural Network (CNN) classifier are investigated to determine optimal phase image exploitation. They all exploit the same Faster-RCNN [25] architecture, but differ in how convolutions are performed. For the first classifier, the input consists of intensity and phase images, and convolutions are performed in real space. This approach, which is classic in image processing, is motivated by the bi-modal nature of the network input.

For the second classifier, the input of the CNN consists of the real and imaginary parts of the electromagnetic field exiting the sample. Those parts are obtained using intensity and phase images. Convolutions are performed in the complex space. This DNN implementation is inspired by existing microscopy approaches, such as dark-field microscopy, which were proven efficient in revealing fine details of the sample. In this case, the microscope objective performs a complex filtering of the electromagnetic field emanating from the sample.

Classifiers’ performances are compared to those obtained with intensity images only. Results reveal that performance significantly improves when phase and intensity images are exploited. This demonstrates the interest of phase modality. We also show that the complex convolution approach is more efficient than real-valued convolution. We believe such behavior is general, and should be investigated for other diagnoses or use cases.

The paper is organized as follows: Materials and methods are presented in Section 2. The principle of the microscopy technique used is introduced (namely Fourier Ptychographic Microscopy, FPM). The malaria infection detection application and the Faster-RCNN detection network architecture are also presented. Section 3 discusses two possible implementations of the bi-modal information of intensity and phase images in the Faster-RCNN. The first relies on real-valued convolution, while the second is attached to complex-valued ones. Section 4 is dedicated to the experimental details, the results, and the discussion. Finally, the conclusion and prospects of this work are detailed in Section 5.

## 2. Materials and Methods

### 2.1. Principle of Fourier Ptychographic Microscopy

Numerous microscopy techniques are available to access the sample phase, in addition to intensity. Among the most important are digital holography [11,26] and computational imaging [27]. Fourier ptychography [28], a recent computational microscopy technique, is used here for its many advantages: it is based on a traditional microscope, and it requires no interferometry that can be delicate to implement. Furthermore, it enables super-resolved images to be obtained using a reconstruction algorithm, with a super-resolution factor γ that can be significant (up to a factor of 6, as demonstrated experimentally).

Finally, it enables images to be reconstructed computationally at different focus planes [29], once the sample has been acquired. Some approaches even enable focus correction to be automatically adapted locally to obtain high-quality images in terms of sharpness and contrast, according to the cell compartments of interest [30]. All these properties make it easy to digitize large image banks, which is necessary to statistically study the contribution of phase images, particularly in the context of the deep-learning framework. From an instrumental point of view, this technique is low-cost [31,32], since a diode array replaces the traditional Köhler illumination source.

**Figure 1:**
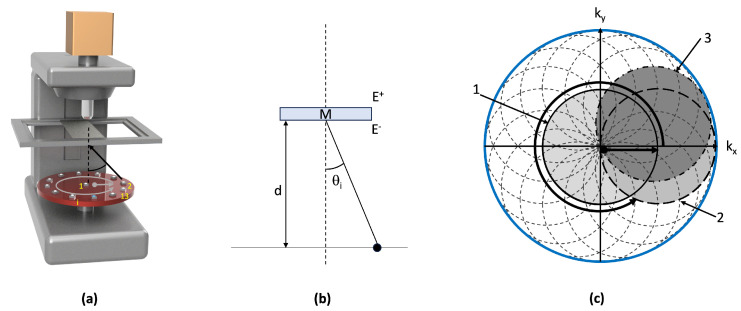
FPM principle. (**a**) Sketch of microscope configuration equipped with its LED matrix, (**b**) illustration of the angular illumination of the sample as determined with spatial position of LED i, (**c**) individual spectral regions acquired by each individual LED. The different raw images captured by the camera are assembled in Fourier Domain with a phase retrieval algorithm. The grayed regions are related to LEDs 1, 2 and 3.

The microscope used is shown schematically in Figure 1a. It consists of an upright microscope equipped with a Plan Apochromat objective lens with a magnification of 10× and 0.45 numerical aperture. The camera used integrates a large 1.1" CMOS sensor with a resolution of 12.34 Mpix of pixel size of 3.45 µm (UI-3200SE from IDS). The camera features a global shutter and an optical area of 14.158 mm × 10.370 mm. The total resolution of the camera is 4104 × 3006 pixels. Similarly to [32,33], the LED array consists of a central diode placed on the optical axis and a ring array of 12 individually controllable LEDs (from Adafruit industry). Each diode is identified by its index *i* with 1≤i≤13; the central diode is numbered 1. The distance d (typically several cm) is such that the illumination at the sample level can be considered coherent on the one hand (Zernike –Van Cittert theorem [34]), and assimilable to a plane wave of wave vector $k_{i}$ on the other hand. In our case, d=30 mm. The wave vector $k_{i}$ is defined as: $|k_{i}|=\frac{2\pi }{\lambda }$ by a far-field approximation. Each diode *i* is thus assigned to a unique illumination propagation direction $u_{i}$ of inclination $\theta_{i}$. λ represents the wavelength used.

Under these conditions, the electromagnetic field immediately beneath the sample that originates from the diode *i* is written as:

(1) $$E_{i}^{-}\left(x,y\right)=A\cdot e^{j(k_{i}^{x}\cdot x+k_{i}^{y}\cdot y)}$$

where A, $k_{i}^{x}$, and $k_{i}^{y}$, respectively, represent its complex amplitude and the projection of the vector $k_{i}$ along the x and y axes. Furthermore, under a thin-sample approximation, the interaction between the light and the sample is modeled by a transfer matrix M(x,y). For the LED *i*, the electromagnetic field immediately above the sample is calculated from M with $E_{i}^{+}\left(x,y\right)=M\left(x,y\right)\cdot E_{i}^{-}\left(x,y\right)$ and the electromagnetic field formed at the camera plane by the microscope objective of the point-spread-function PSF(*x*, *y*) is:

(2) $$E_{i}^{\mathrm{cam}}\left(x,y\right)=\left[M\left(\frac{x}{G},\frac{y}{G}\right)\cdot E_{i}^{-}\left(x,y\right)\right]*\mathrm{PSF}\left(x,y\right)$$

where *G* represents the magnification of the microscope objective and ∗ is the convolution product, as in [35]. In the spectral domain, $E^{\mathrm{cam}}$ is expressed as:

(3) $${\hat{E}}_{i}^{\mathrm{cam}}\left(k^{x},k^{y}\right)=A\cdot \hat{M}\left(G\left(k^{x}-k_{i}^{x}\right),G\left(k^{y}-k_{i}^{y}\right)\right)\cdot \mathrm{CTF}\left(k^{x},k^{y}\right)$$

where CTF denotes the coherent optical transfer function of the microscope objective (the Fourier Transform of PSF). For a lens that is aberration free, the $CTF\left(k^{x},k^{y})\right)$ function is equal to 1 inside a disk D of radius $r=2\pi \cdot \frac{NA}{\lambda }$ centered at $\left(0,0\right)$ and zero elsewhere. Here, NA denotes the objective’s Numerical Aperture and ^ denotes the function in the Fourier domain. The ith recorded image $I_{i}$ is then associated with the following equation:

(4) $$I_{i}\left(x,y\right)={\left|E_{i}^{\mathrm{cam}}\left(x,y\right)\right|}^{2}$$

This last equation corresponds to the forward problem relative to the image formation of the sample with the ith LED. Examination of Equations (3) and (4) reveals that the region of the object spectrum imaged with diode *i* corresponds to the spectral region of M bounded by the $\left(k_{i}^{x},k_{i}^{y}\right)$ translated disk D. For illustrative purposes, the regions associated with the LEDs 1, 2 and 3 are shown in Figure 1c in gray. The calculated overlap factor Γ between the regions associated with two successive LEDs is of 67% (except for LEDs 1 and 2, for which Γ=39%). The dashed circles indicate the regions of the spectrum of M paved by the different diodes of the illumination matrix. Note that the total area covered (blue circle) is much larger than that accessible through the microscope’s numerical aperture. This corresponds to the aperture synthesis mechanism used in FPM to obtain super-resolved images. Such aperture synthesis is possible, provided the raw images are adequately assembled in the spectral domain and the phase information lost during the measurement process (cf. Equation (4)) is retrieved. Different methods are available such as ePIE [36] or EPRY [37] algorithms. If we denote $\tilde{M}$ the guessed solution and ${\tilde{I}}_{i}$, the image attached to $\tilde{M}$ for LED *i* obtained after applying the direct model, then:

(5) $$\sqrt{{\tilde{I}}_{i}\left(x,y\right)}=\left|F^{-1}\left(\hat{\;\tilde{M}\;}\left(k^{x},k^{y}\right)*\mathrm{CTF}\left(k^{x}-k_{i}^{x},k^{y}-k_{i}^{y}\right)\right)\right|$$

This image corresponds to the one that the camera would measure if the complex mask describing the sample was $\tilde{M}=F^{-1}(\hat{\tilde{M}})$. As a result, the researched solution is found by minimizing the error function:

(6) $$L=\sum_{i}{\left|\sqrt{{\tilde{I}}_{i}\left(x,y\right)}-\sqrt{I_{i}\left(x,y\right)}\right|}^{2}$$

The algorithms mentioned above proceed iteratively, and $\tilde{M}$ is reconstructed progressively with descent-gradient calculations. These calculations exploit images $I_{i}$, updating the spectral region associated with LED *i* and iterating *i*. After convergence, super-resolved intensity and phase images are extracted, calculating the inverse Fourier transform of $\tilde{M}$.

### 2.2. Malaria Application

Among the various methods that have been explored for malaria diagnosis, microscopy analysis remains the gold standard technique. Traditionally, the microscopic malaria diagnosis is obtained after a multi-step approach involving collecting a blood sample and conducting two main examinations. The thick blood smear is utilized to detect the presence of parasites. If its the case, the thin blood smear is examined under a microscope to provide a detailed analysis of parasite morphology, identify the species, and determine parasitemia. These diagnostic steps are typically performed manually by healthcare professionals and can be time-consuming, especially when dealing with a high volume of patients.

Scientists have looked into various automation and diagnostic support methods to tackle the tedious task of examining thick and thin blood smears for parasite detection. The constraints are high, since a detection with a high sensitivity is required as well as correct species identification. Recent advancements in computer vision, particularly in deep learning algorithms, have shown promise in detecting *Plasmodium Falciparum* parasites [38,39,40,41,42,43,44,45]. Although considerable progress has been made, the various methods developed to date are not sufficiently effective when parasitemia is low and parasites are tiny.

Let us consider the case of *Plasmodium Falciparum*, the causative agent of malaria’s most severe and potentially fatal form. During the early stage of infection, parasitemia can be quite low. As a result, if one wants to diagnose malaria from only a thin blood smear (eliminating the thick blood smears step), it becomes necessary to examine a significant number of red blood cells (at least 200,000) to attain the required sensitivity. This can be particularly demanding when considering the required resolution and limitations imposed by the microscope objective’s numerical aperture. A high NA (typically around 0.9–1) is needed to achieve the nanometric resolution necessary for precisely detecting parasites. However, a larger NA also means a smaller field of view. As a result, thoroughly examining the area of good spreading, approximately 25 mm^2^, would require considerable time for a highly trained operator.

**Figure 2:**
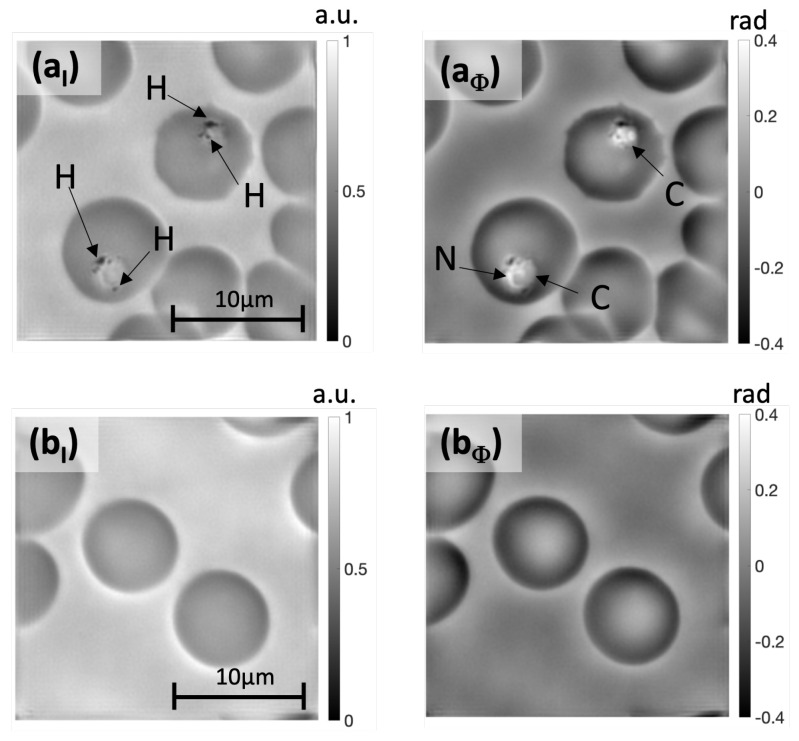
Illustrative images of stained blood smears obtained with FPM after reconstruction. (**a_I_**) Intensity and (**a_Φ_**) phase images of two red blood cells parasitized with *Plasmodium falciparum*. In the intensity image, hemozoin pigments are indicated with the H arrow. Details of parasite structures hardly visible in the intensity image such as nuclear material N and cytoplasm C are revealed in the phase image. For comparison, intensity and phase images of healthy red blood cells are presented in (**b_I_**,**b_Φ_**) respectively.

FPM is an alternative approach to address the challenge of studying a large number of blood cells in a reasonable time. Indeed, FPM provides a more favorable balance between resolution and field of view size than conventional microscopy techniques. Moreover, intensity and phase offer complementary information that helps to characterize the parasite and red blood cells precisely as shown in Figure 2. Indeed, in addition to the compartments of the parasite visible in the intensity image **a_I_**, the phase image **a_Φ_** shows the nuclear material (N arrow) in white and the cytoplasm (C arrow). Additionally, the phase **b_Φ_** shows the hollow center of the red blood cell not visible on the intensity image **b_I_**.

### 2.3. Detection Model

Similarly to [42], we have chosen a network model allowing the joint location and classification of red blood cells into parasitized or healthy. In this manner, numerous cells can be processed all at once from a single large field of view. Among the different models in the literature for such detection tasks, a Faster-RCNN [46] has been chosen in this study. Indeed, it is known for generating regional bounds with minimal time cost compared to older versions of RCNN [25,47]. Faster-RCNN has also been proven to be very accurate in general and specifically adapted to small object detection in comparison to other models such as Yolo [48,49].

**Figure 3:**
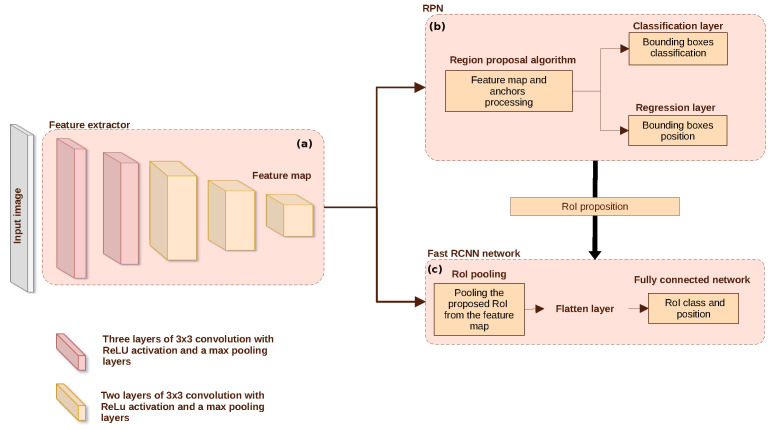
The architecture and different modules of the Faster-RCNN model: (**a**) the feature maps extraction module, (**b**) the region proposal networks modules, and (**c**) the Fast-RCNN networks module for classification.

Its architecture is presented in Figure 3; it relies on three modules: two decision networks (b) and (c), connected through a feature extractor (a). An image is given as an input to the model and processed by the feature extractor layers Figure 3a. Here, the feature extractor is based on VGG-16 architecture [50], which is a succession of 16 layers of 3 × 3 convolutions with ReLU activation functions and pooling layers. A feature map is then produced and transmitted simultaneously to modules (b) and (c).

Module (b) corresponds to the Regional Proportional Network (RPN). The RPN is responsible for detecting the objects of interest within an image. It generates a set of candidates, which are potential bounding boxes that might contain objects of interest. The RPN proposes bounding box coordinates and their associated likelihood to contain an object of interest. This is achieved by a set of pre-defined anchors, which are fixed-sized boxes, placed at various positions and scales across the feature map. The set of bounding boxes is sent to the second branch (module (c)) along with the feature map resulting from the module (a).

In module (c), the corresponding region of the bounding box is pooled from the feature map and sent to the Fast-RCNN network to predict its class and give its final coordinates.

The model is trained alternatively between the RPN and the Fast RCNN modules. First, one performs one iteration of the RPN and retro-propagates the error from (b) to (a). Then, the proposals from RPN are used to train the Fast RCNN, and the error is propagated from (c) to (a). The network tuned by Fast RCNN is then used to reinitialize RPN, and this process is iterated.

The specifics of the architecture are detailed in Table 1.

## 3. Bi-Modal Image Exploitation in Convolutional Neural Networks

As explained in the previous section, the data from the QPI device is bi-modal in that it corresponds to two (Intensity-Phase) images resulting from the FPM reconstruction process. Different possibilities for representing this data can be considered. Either intensity and phase can be seen as two independent real-valued matrices (in this case, we speak of a real-valued representation of the bi-modal information), or the values of intensity and phase can be represented as a single matrix of complex numbers (in that case, we speak about complex-valued representation).

This offers different exploitation possibilities in neural networks. For real-valued representation, real-valued operations are generally employed leading to real-valued neural networks (RVNN). They efficiently integrate information from multiple sources and leverage the capacity to fuse diverse data modalities for optimal classification performance. This approach exploits two distinct channels, where intensity and phase represent separate information emerging from distinct physical processes.

In the case of complex-valued representations, we can envisage operating directly on complex numbers in what is called a complex-valued neural network (CVNN). This CVNN has been extensively examined in the literature, covering both implementation and theoretical aspects. For instance, [51] presents proof of the approximation theorem for complex-valued neural networks. In [52], researchers investigate the blocks comprising real-valued neural networks and their counterparts in CVNNs. Mathematical and empirical studies have been conducted on back-propagation, cost function selection, and activation functions for this type of network in [52,53,54,55].

CVNNs have been implemented in several applications such as the classification of MRI data [56,57], acoustic signal processing [58], and seismic data analysis [53]. In those fields, where the phase information is obtained from a direct measure, CVNNs exhibit smaller errors and yield better generalization abilities than their real-valued counterparts. However, CVNNs have not yet been applied to the classification of bi-modal intensity-phase images produced by the FPM. In this microscopy technique, phase results from a computational reconstruction rather than a direct measure. It is, therefore, interesting to implement CVNNs for our use case and compare them to real-valued counterparts.

In CVNNs, real-valued convolution must be replaced by complex-valued convolution. In this case, the representation of complex data using the real and imaginary parts offers the possibility to apply this type of filtering using common neural network implementations. However, this filtering also introduces modifications to the operations used, including cost functions and other aspects of the network.

We detail the differences in implementing real versus complex convolution in CNNs in the following sections.

### 3.1. Real-Valued Convolution

One of the key operations in CNNs is convolution. Real-valued convolution is used to extract meaningful features from the input data while preserving the spatial relationships and patterns present in the data.

**Figure 4:**
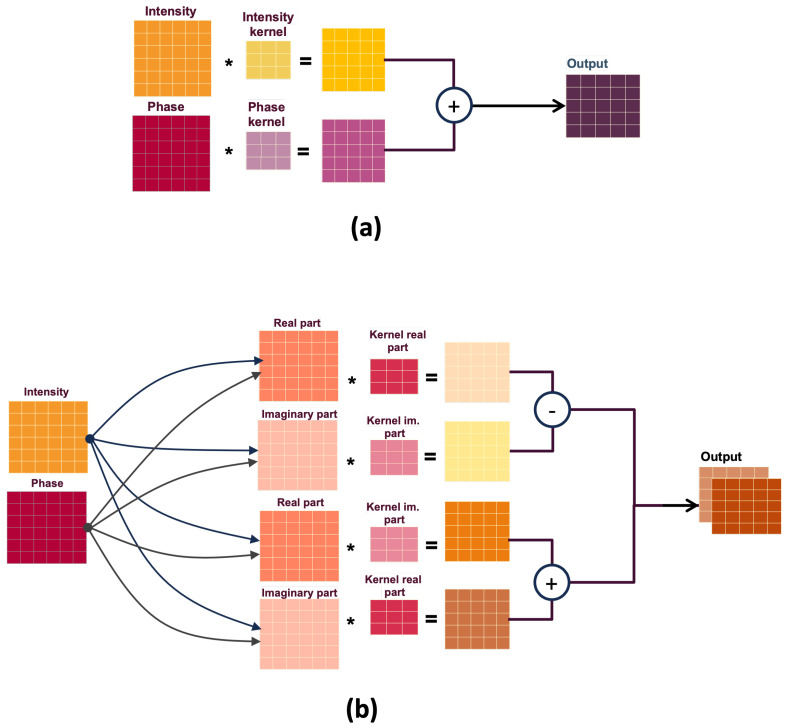
Schematic representation of the filtering operation in a real-valued implementation (**a**) versus a complex-valued one (**b**).

Figure 4a illustrates the principle of real-valued convolution when applied to our case; each component of the input image, here intensity and phase, is processed independently by its corresponding kernels. The intensity component is convoluted with intensity kernels, and the phase component is convoluted with phase kernels. In this way, distinct characteristics are extracted for each channel individually.

However, after the individual convolutions, the resulting filtered images are mixed to form the final output, where the corresponding pixel values of the different filtered images are added together to create a single output channel. In this manner, the neural network aggregates both information adaptively, depending on the task.

In summary, the two physical processes in the system are treated as separate channels during the convolution process, allowing specific features to be extracted independently from each channel. This independence ensures that the unique information from both processes is preserved and adaptively merged. As a result, the final output benefits from the complementary nature of the two independent physical processes.

### 3.2. Complex-Valued Convolution

While real-valued convolutions operate on real numbers, complex convolutions consider inputs as complex numbers. Complex convolutions use complex numbers as coefficients in the filters to capture the rich interaction between amplitude and phase.

Since standard deep-learning libraries can only handle real numbers natively, some adaptation in the implementation of the convolution block of the network is required, as depicted in Figure 4b. Complex numbers are represented as pairs of real numbers (real and imaginary parts) so the operation may be adapted to the existing implementations (Tensorflow, PyTorch) as described below. The idea is to introduce the complex domain constraints in the formalism usually exploited for real-valued convolution. The changes will therefore concern the representation of the filter weights and the input by using a bi-modal (real, imaginary) representation and redefining the convolution operation.

More precisely, one can define the complex convolution using only real values while considering the particularities of the four arithmetic operations. The complex-valued input *M* is written as $M=(M_{R}+iM_{I})$. The complex convolution filter *K* is given by $K=(K_{R}+iK_{I})$. This is equivalent to defining two real-valued filters, where one represents the real part and the other the imaginary part of the complex filter (see Figure 4). The convolution of *M* by *K* is written:

(7) $$\begin{array}{cc} K*M & =\left(M_{R}+iM_{I}\right)*\left(K_{R}+iK_{I}\right)\\ \; & =\left(M_{R}*K_{R}-M_{I}*K_{I}\right)+i\left(M_{R}*K_{I}+M_{I}*K_{R}\right) \end{array}$$

A tensorial representation can be given by:

(8) $$\left[\begin{array}{c} R(M*K)\\ I(M*K) \end{array}\right]=\left[\begin{array}{cc} K_{R} & -K_{I}\\ K_{I} & K_{R} \end{array}\right]\left[\begin{array}{c} M_{R}\\ M_{I} \end{array}\right]$$

We note that the conventional activation functions commonly used in neural networks are unsuited for complex-valued data due to their reliance on real number operations, particularly the maximum (max) function, such as Rectified Linear Unit (ReLU). The other activation functions are also unsuitable for complex numbers; Sigmoid or Tanh have singularities for some complex numbers values (iπ(2N−1)).

Several choices have been made in the literature to design activation functions adapted to learning from complex data [55]. One of them, defined as the extended version of ReLU for complex-valued data, called CReLU, has been proposed and tested in [52], which reports that CReLU is gives the best performances in comparison to other functions like Sigmoid, which seems unstable. The complex activation CReLU applies separate ReLU on both the real and the imaginary part of a neuron, i.e.:

(9) CReLU(z)=ReLU(R(z))+iReLU(I(z))

where R(.) and I(.) are the real and the imaginary parts, respectively.

This activation function will be used in our complex-valued architecture. This function allows us to separately (or jointly, when both real and imaginary parts are negative) cancel the real and the imaginary part when they are negative and only preserve the most relevant extracted information.

The pooling operation of CNNs must also be adapted to CVNNs. This operation aims to retain the most prominent features; it is performed by calculating the maximum value of the set of patches composing the feature map in order to downsample it. Currently, max pooling is employed in the majority of CNNs, since it provides numerical stability during training and it allows a reduction in the feature map size. Still, this operation is not defined for complex-valued data. A solution would be to apply the max pooling separately to the real and the imaginary channel, which can dismiss the mathematical correlation between the two parts. Instead, we use an average pooling operation in our CVNNs, since it is well-defined in the complex domain.

## 4. Experimental Evaluation

### 4.1. Dataset

A set of thin blood smears from nine patients was scanned using the FPM microscope configuration described above. The surface of the good spreading is completely scanned with 16 Fields Of View (FOVs). For each acquired FOV, 13 raw images were acquired, each corresponding to a LED with a specified lighting angle. The reconstruction of the phase-intensity bi-modal images is then performed using the E-Pie algorithm [36] applied to the 13 acquisitions. The reconstructed images are then cropped into smaller bi-modal images, such that each image contains approximately 200–300 red blood cells. An expert labeled all the fields by indicating the position of each red blood cell with a box around the cells and by providing a label indicating the presence or absence of a parasite. The final dataset contains 2216 bi-modal intensity-phase images of 896×896 pixels, presenting 418,389 healthy and 65,140 infected cells.

Although the database is limited in the number of patients, it should be noted that the images contain parasites of different sizes and that the infection levels vary between 1 and 5%, which is representative of actual use cases in malaria diagnosis.

### 4.2. Implementation Design

The intensity and phase images resulting from the FPM reconstruction are explored using real and complex-valued representations in a Faster-RCNN. The objective is to detect the red blood cells and classify them into parasites or healthy cells. More precisely, three Faster-RCNN models for *Plasmodium Falciparum* parasites detection were implemented; all models have the same number of trainable parameters:

- A classical real-valued Faster-RCNN using intensity only (I-RV);
- A classical real-valued Faster-RCNN using intensity and phase (I/ϕ-RV);
- A complex-valued convolution Faster-RCNN using intensity and phase (I/ϕ-CV).

The complex-valued Faster-RCNN is obtained from the real-valued architecture shown in Figure 3 by replacing the convolutions in the shared branch (Module (a)) with complex-valued convolutions, changing the activation function to CReLU and average pooling as described in Section 3. Several key hyper-parameters in the Faster-RCNN architecture have been tuned. The search for the best hyper-parameters for each network was carried out in such a way as to have an optimal classification performance for each experience. Moreover, we noticed that the maximum number of boxes predicted by the RPN greatly influences the rate of well-detected parasitized cells (Positive Rate). Indeed if a small number of boxes is chosen, several cells are not detected, which gives a low positive rate, knowing that most cells are healthy. Contrariwise a very high number of boxes leads to certain confusion (several boxes are predicted for one object), degrading the classification quality. Among others, the anchors used in the RPN are the most influential parameters, which have to be chosen considering the variability in blood cell sizes between patients and the intersection over the union (IoU) threshold for overlapping boxes.

The models’ implementation was performed using TensorFlow version 2.1 and trained using NVIDIA Corporation TU102GL Quadro RTX 6000/8000 GPU and Adam optimizer with a learning rate $10^{-4}$. The code is available from [59].

The data must be normalized to ensure fast and efficient learning in the chosen architecture. Here, a normalization adapted to each representation was carried out. In the case of the real-valued network, each channel has been normalized using a min-max normalization applied to each channel separately to ensure the information can be mixed and extracted consistently. In the case of complex networks, the min-max normalization is performed on the module of the data to guarantee the preservation of the relationship between the module and the phase and, thus, preserve its physical meaning.

To compare the performance of the three implementations, we use two criteria, the True Positive Rate (*TPR*) and the True Negative Rate (*TNR*) of parasite detection. This is performed on the same databases and with the same protocols.

*TPR* and *TNR* result from the definitions of *TP*, *TN*, *FP*, and *FN*, which were adapted slightly to our specific *Plasmodium falciparum* detection task as follows:

(10) $$TPR=\frac{TP}{P}=\frac{TP}{TP+FN}\;TNR=\frac{TN}{N}=\frac{TN}{TN+FP}$$

- True positive (*TP*) is the number of well-classified parasitized red blood cells;
- True negative (*TN*) is the number of well-classified healthy red blood cells, the extra healthy cells and missed healthy cells;
- False positive (*FP*) is the sum of the wrongly classified healthy red blood cells and the extra parazited blood cells;
- False negative (*FN*) is the sum of the wrongly classified parasitized red blood cells and the missed parasites.

### 4.3. Results and Discussion

The parasite detecting results for the different implementations are provided in Table 2 and Table 3.

The details of the output boxes for each model are shown in Table 2. To construct the table, a localization threshold of 0.7 was used. This means that a predicted bounding box is considered correctly positioned if it overlaps with the corresponding ground truth box by at least 70%. If the predicted label matches the ground truth label, the cell in this box is considered well-classified by the model. Otherwise, the cell is considered misclassified. The missed cells regroup the boxes containing cells that were not predicted by the model. Lastly, the model’s added boxes are the boxes that have poor overlap with ground truth or boxes that do not correspond to actual objects.

The first observation in Table 2 is that introducing phase information reduces the misclassification of infected and healthy cells. It also reduces the number of healthy and infected missed cells.

In the context of model performance comparison, I/ϕ-CV seems to be the most efficient for the task. This model achieves the highest count of well-classified infected cells and healthy cells, and the lowest count of misclassified infected cells. This suggests that the integration of the complex feature extractor enhances the classification abilities of Fast-RCNN module (referred to in Figure 3). Furthermore, the model also achieves the lowest count of missed cells for both healthy and infected categories. These results indicated an augmentation in the abilities of RPN module.

From Table 3, we observe at a high *TNG* (the red blood cells are well detected and well labeled), and a *TPR* varying between 93,33% to 97,15%. These values are promising, as the main goal in *Plasmodium Falciparum* detection is to obtain a high *TPR* to detect cases of early-stage infection, where parasite cells are extremely sparse among the total red blood cells. The confidence intervals mentioned in Table 2 are obtained thanks to a cross-validation method, which is widely used in machine learning model evaluation. More precisely, in this work, we used a k-split cross-validation algorithm [60], as it offers a better estimation of the model’s generalization ability by performing not one but k measures of the validation metric. Its principle is simple: The data sample is divided into k smaller sample datasets. k independent evaluations of the model are then made, and at each evaluation, a single subsample is retained for testing the model, while the remaining subsamples are used for training. The experiments’ results are averaged, and the standard deviation is computed to estimate each evaluation metric robustly. Here, we chose a five-fold cross-validation where the original dataset was partitioned the five equitably sized subsets (roughly 443 samples per subset).

Two significant conclusions can be driven from Table 3. The first concerns the impact of using both intensity and phase images compared to intensity images alone (I/ϕ-RV and I/ϕ-CV compared to I-RV). The experiments show that for an equivalent value of *TNG*, the sensibility is increased by 2% when using I/ϕ-RV, and by 4% when using I/ϕ-CV, which means that more parasites are rightly detected. It, therefore, illustrates the contribution of phase information to improve the quality of diagnosis of rare events. In this case study, we observe that the phase image brings complementary information to intensity, even in the case of non-transparent objects, where the phase is usually considered to have a minor effect. It can therefore provide crucial information for analyzing microscopic images, paving the way toward efficient automatic systems for malaria detection.

The other outcome of our experiments concerns the relevance of the complex implementation towards the real one for the intensity-phase images. A significant improvement of roughly 2% in *TPR* is obtained with the complex formalism compared to the real one. Moreover, the confidence intervals that the network provides with complex formalism (I/ϕ-CV compared to I/ϕ-RV) are narrower than in the other experiments. This result, coupled with the mean *TNG* and *TPR* values, highlights the superior generalization capabilities of complex-valued neural networks.

We statistically validate this performance difference between the real and complex-valued models using the McNemar test [61,62]. For each fold, the *p*-value was lower than 0.001, which means we reject the null hypothesis (the two models have the same error rate). This indicates that the model with complex formalism is doing better and that the marginal proportions are significantly different, confirming the pertinence of the performance results.

Moreover, our results show that even a partial complex implementation (the complex formalism is implemented only in the first part of the model to find the feature map that will be further used in branches (b) and (c) (referred to in Figure 3), allows a performance improvement.

**Figure 5:**
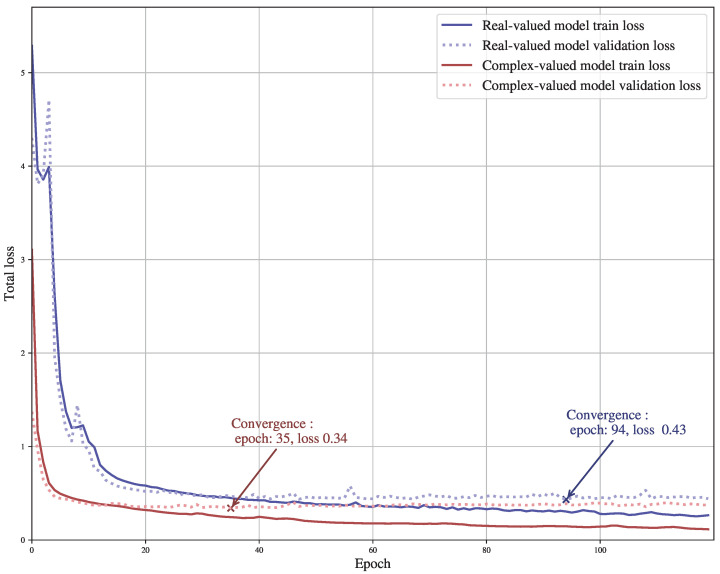
Train and validation loss curves for complex-valued and real-valued models.

Another interest of the complex CNN implementation is that it allows for faster convergence. Indeed, one can remark in Figure 5, which shows the total loss for each epoch of each model, that the real-valued model reaches its lowest loss value (0.43) on the validation database after 94 epochs. In comparison, the complex-valued one requires only 35 epochs to reach the best validation point, with a lower loss value (0.34). Those convergence points are indicated in Figure 5 by a cross.

We recall the motivations behind the two proposed exploitations of intensity and phase images: on the one hand, the parameters related to the light-matters interactions (absorption, optical path) are explored for the real-valued convolution approach, while on the other hand, the image associated with matrix transfer (itself related to the EM field emanating from the sample) is filtered in a complex manner using the complex-valued implementation.

This is why, from a physical point of view, both complex and real filtering for extracting relevant features from intensity and phase data are justifiable. The performance improvement that we measure with the complex model can therefore be interpreted by the efficiency of the complex formalism (both in terms of input coding and complex filtering). This is, in particular, confirmed by the reduction in the confidence intervals observed with complex filtering in Table 3. This indicates greater precision, reliability, and tighter generalization capabilities. These results are consistent with the state of the art in different application areas, as already mentioned. This complex implementation is, therefore, legitimate when bi-modal data are available. Moreover, as mentioned by other authors, this implementation allows the network to have a richer representational capacity and thus improves the performance of our detection application.

Note that, in previous studies, both intensity and phase result from the acquisition device (such as MRI or radar) and improvement is observed with a complex implementation. The application considered in this paper is not exactly similar, as the phase is not measured; it results from a computational reconstruction, but the same conclusions can still be drawn.

## 5. Conclusions

In this paper, we proposed a novel workflow for efficient malaria detection relying on FPM imaging of thin stained blood smears. It exploits a couple of bi-modal images (intensity and phase) using complex-valued neural networks. We considered the case of parasite detection in red blood cell images, and showed that using joint intensity and phase information, even for stained objects, leads to better performance than using only intensity images. This result reveals that the phase information is complementary to the intensity, even for stained objects for which intensity images could be considered sufficient. Also, the complex-valued formalism is shown to be more efficient than the real-valued one for processing FPM bi-modal data. These promising results should be confirmed using larger datasets and other case studies. Moreover, limiting the complex-based implementation to the convolution part of a DNN is sufficient to benefit from the bi-modal, complex nature of the information. This point is interesting, as it will allow generalization to many deep network models which use convolution in their first stage for feature extraction. We, therefore, believe that this work should easily be extended to other QPI imaging techniques and applications, relying on various deep network architectures.

## Figures and Tables

**Table 1:** Parameters of Faster-RCNN architecture as shown in Figure 3.

Module	Layer	Input Shape	Output Shape	Kernel Size
Feature extractor	Convolution	(896, 896, 2)	(896, 896, 64)	(3, 3)
	Convolution	(896, 896, 64)	(896, 896, 64)	(3, 3)
	MaxPooling	(896, 896, 64)	(448, 448, 64)	(2, 2)
	Convolution	(448, 448, 64)	(448, 448, 128)	(3, 3)
	Convolution	(448, 448, 128)	(448, 448, 128)	(3, 3)
	MaxPooling	(448, 448, 128)	(224, 224, 128)	(2, 2)
	Convolution	(224, 224, 128)	(224, 224, 256)	(3, 3)
	Convolution	(224, 224, 256)	(224, 224, 256)	(3, 3)
	Convolution	(224, 224, 256)	(224, 224, 256)	(3, 3)
	MaxPooling	(224, 224, 256)	(112, 112, 256)	(2, 2)
	Convolution	(112, 112, 256)	(112, 112, 512)	(3, 3)
	Convolution	(112, 112, 512)	(112, 112, 512)	(3, 3)
	Convolution	(112, 112, 512)	(112, 112, 512)	(3, 3)
	MaxPooling	(112, 112, 512)	(56, 56, 512)	(2, 2)
	Convolution	(56, 56, 512)	(56, 56, 512)	(3, 3)
	Convolution	(56, 56, 512)	(56, 56, 512)	(3, 3)
	Convolution	(56, 56, 512)	(56, 56, 512)	(3, 3)
RPN	Convolution	(56, 56, 512)	(56, 56, 512)	(3, 3)
	Convolution	(56, 56, 512)	(56, 56, 9×4)	(1, 1)
	Convolution	(56, 56, 512)	(56, 56, 9)	(1, 1)
Fast RCNN	RoI proposition function	-	(1500, 4)	-
	RoI Pooling function	-	(7, 7, 512)	-
	Flattening layer	(7, 7, 512)	(25088)	-
	Fully connected	(25088)	(4096)	-
	Dropout (0.5)	(4096)	(4096)	-
	Fully connected	(4096)	(4096)	-
	Dropout (0.5)	(4096)	(4096)	-
	Fully connected	(4096)	(4×3)	-
	Fully connected	(4096)	(3)	-

**Table 2:** Detailed results of Faster-RCNN predictions averaged to the nearest unit over the five folds.

	I-RV	I/ϕ-RV	I/ϕ-CV
Well-classified infected	12,116	12,320	12,670
Misclassified infected	528	433	324
Well-classified healthy	81,818	82,778	82,965
Misclassified healthy	551	295	372
Missed infected	356	186	46
Missed healthy	1086	601	169
Added infected	127	154	119
Added healthy	347	554	620

**Table 3:** Experiments evaluation metrics.

	I-RV	I/ϕ-RV	I/ϕ-CV
TNR	99.18 ± 0.20%	99.34 ± 0.24%	99.10 ± 0.07%
TPR	93.33 ± 1.33%	95.22 ± 0.73%	97.15 ± 0.30%

## Data Availability

The data are not rendered publicly available due to privacy restrictions.
